# Analysis of the Clinical Application Value of Immune Function Markers and Cytokines in the Recurrent Aphthous Ulcer

**DOI:** 10.1002/cre2.70418

**Published:** 2026-07-23

**Authors:** Yiwen Chen, Ting Meng, Lahong Zhang, Qiang Ke

**Affiliations:** ^1^ Clinical Laboratory Department Hangzhou Normal University Affiliated Hospital Hangzhou Zhejiang China; ^2^ Medical Laboratory Techology School Wengzhou Medical University Renji College Wenzhou Zhejiang China

**Keywords:** cytokine, immune function, recurrent aphthous ulcer

## Abstract

**Background:**

This study aimed to analyze the alterations in cellular and humoral immune functions, alongside cytokine profiles, in patients with recurrent aphthous ulcer (RAU) compared to those with other oral mucosal diseases and healthy controls (HC), and to investigate their clinical significance.

**Methods:**

Anticoagulated whole blood and serum samples were collected from 101 cases of the RAU group, the “Others” group, and the HC group. The percentages of lymphocyte subsets, including NKT, NK, CD4^+^T, CD8^+^T, and CD3^−^CD19^+^B cells, were detected by flow cytometry. The concentrations of IgG, IgA, IgM, IgE, C3, and C4 were measured using immunoturbidimetry. Additionally, the levels of cytokines IL‐6, IL‐8, and TNF‐α were quantified by chemiluminescence. Statistical analyses were performed to compare the clinical value of these functional markers across the different groups.

**Results:**

Non‐parametric tests revealed that the percentage of NKT cells in the RAU group was significantly higher than those in both the “Others” and HC groups; furthermore, the “Others” group showed significantly higher levels than the HC group. The content of IgE in the RAU group was significantly elevated compared to the “Others” and HC groups. The levels of IL‐6, IL‐8, and TNF‐α in the RAU group were significantly higher than those in the HC group. ROC analysis revealed that NKT cells, IgE, IL‐6, and IL‐8 showed AUC values greater than 0.7 in the diagnosis of RAU.

**Conclusion:**

This study demonstrates that the combined detection of NKT cells, IgE, and cytokines (IL‐6, IL‐8, and TNF‐α) may potentially hold significant clinical application value in the assessment of RAU and other oral mucosal diseases.

## 1 Introduction

Recurrent aphthous ulcer (RAU) is the most prevalent disorder of the oral mucosa, affecting at least 10%–25% of the general population. Characterized by painful ulceration and frequent recurrence, it is difficult to achieve a complete cure for this condition, thus significantly impacting patients' quality of life and daily functioning. Although the exact etiology of RAU remains incompletely understood (Chinese Stomatological Association Oral Mucosal Diseases Committee 2012), its pathogenesis is known to involve a multifactorial interplay of immunity, infection, genetics, and environmental factors. In particular, cellular immune responses play a pivotal role in the disease process. Consequently, the immunological pathogenesis of RAU has attracted extensive attention (Chinese Stomatological Association Oral Mucosal Diseases Committee 2012; Anxun 2021), and the assessment of immune function holds significant clinical value for the diagnosis and management of various oral mucosal diseases.

All immune responses are orchestrated by complex networks involving lymphocytes, monocytes, macrophages, and their associated cytokines. Research indicates that the quantity and function of lymphocyte subsets—including B cells, T cells, and NK cells—are closely correlated with the disease status and progression of RAU. NKT cells represent a distinct T cell subset co‐expressing markers of both T cells and natural killer cells. The intrinsic cytotoxicity of NKT cells, coupled with their regulatory effects on other immune cells—which can either suppress or sustain inflammation—highlights their pivotal role in the pathogenesis of RAU. However, studies focusing on their role in RAU remain limited. Cytokines are a class of small soluble proteins secreted by immune cells and histiocytes that play a pivotal role in immune responses, inflammation, and tissue repair (Hua et al. 2020; Lilin et al. 2017).

This study analyzed the percentages of NKT cells and other lymphocyte subsets, along with the levels of immunoglobulins, complement components, and cytokines (IL‐6, IL‐8, and TNF‐α) in patients with RAU and other oral mucosal diseases. The objective was to explore the clinical value of immune function assessment in these conditions and to establish a research foundation for elucidating the immunological pathogenesis of RAU.

## 2 Materials and Methods

### 2.1 Sample Collection

A total of 101 untreated RAU patients, 101 patients with other oral mucosal diseases (Others group), and 101 healthy controls (HCs) admitted to the Affiliated Hospital of Hangzhou Normal University between October 2023 and November 2023 were enrolled as study subjects. No significant differences in age or gender were observed among the three groups (Table 1).

**Table 1:** Demographic characteristics of the patients.

Characteristic	RAU	Others	HCs	*p* value
Age	44.3 ± 15.2	50.3 ± 15.4	43.6 ± 8.5	*p* > 0.05^a^
Sex				
Male	65 (64.3%)	63 (62.4%)	58 (57.4%)	*p* > 0.05^b^
Female	36 (35.6%)	38 (376%)	43 (42.6%)	*p* > 0.05^b^

^a^ *p* values were calculated using the Mann–Whitney test for continuous variables.

^b^ *p* values were calculated using Fisher's exact test for categorical variables.

Lymphocyte subset and activation Indicator, immunoglobulins, and complement levels were assessed across all 101 samples from the three experimental groups. However, because cytokine assays were conducted retrospectively as a supplementary experiment, the final analysis was restricted to 40 paired samples, with comparative evaluations limited exclusively to the RAU and healthy control groups.

### 2.2 Inclusion Diagnostic Criteria

Patients in the RAU group were clinically diagnosed with RAU based on the following criteria: a frequency of ≥ 3 episodes per year; presentation for treatment within 48 h of ulcer onset; and no systemic or topical drug therapy within 1 month prior to the study. All participants demonstrated adequate cognitive and communicative abilities.

The Others group comprised 53 patients with oral lichen planus, 21 patients with ulcers unrelated to RAU, 15 patients with angular stomatitis, and 12 patients with chronic periodontitis.

The healthy control group (HCs) consisted of individuals undergoing routine physical examinations at the same hospital during the same period, with no history of oral disease.

Exclusion criteria included the presence of systemic inflammatory responses, autoimmune diseases, oral malignancies, or organic disorders such as hepatic, renal, or cardiac dysfunction.

### 2.3 Lymphocyte Subset and Activation Indicator Detection

Samples of EDTA‐anticoagulated peripheral blood (5 mL) were collected from the patients of the three groups. Fifty microliters of whole blood was placed in a Trucount tube and 20 μL of fluorescent antibodies (anti‐CD3−FITC, anti‐CD4‐PE, anti‐CD8‐APC, anti‐CD19‐PE, anti‐CD16‐FITC, and anti‐CD56‐APC, BD Company, USA) were added to it; the above mixture was placed away from light for 20 min, and then 450 µL of hemolysin was added to it and allowed to stand under the same conditions for 15 min before testing on the machine. The percentages of CD3+/CD4+/CD8+ T cells, CD3−CD19 + B cells, CD3−CD16+CD56+ NK cells, and CD3+CD16+CD56+ NKT cells were measured using multi‐color flow cytometry. Samples were analyzed on a flow cytometry system (Becton Dickinson, FACSCanto II, USA) according to standard operating procedures, and absolute counts were calculated.

### 2.4 Immunoglobulins' (IgA, IgG, IgM, and IgE) and Complements' (C3 and C4) Detection

Peripheral blood samples (8 mL) were collected from patients in the three groups using coagulant tubes. Serum was separated by centrifugation for analysis. The serum samples were stored at −80°C. Immunoglobulins (IgA, IgG, IgM, and IgE) and complement components (C3 and C4) were quantified using a specific protein immunoturbidimetric analyzer (Beckman Coulter, IMMAGE 800, USA). The assay used a rate nephelometry method, which measures the rate of scattered light intensity resulting from the formation of antigen–antibody complexes.

Specific procedure: the prepared serum sample cup was placed in the instrument's sample rack, and the sample number and corresponding parameter (e.g., IgG and C3) were input into the computer system. Reaction process (automatically executed by the instrument): The instrument probe aspirates the sample (approximately 2–5 μL) and mixes it with reagent R1 (buffer solution), then incubates the mixture at 37°C for 5 minutes to eliminate sample background interference. Reagent R2 (antibody‐coated latex particles) was added and mixed thoroughly. At 37°C, the instrument continuously monitored absorbance changes at specific wavelengths (e.g., 340 and 840 nm).

### 2.5 Cytokines (IL‐6, IL‐8, and TNF‐α) Detection

Serum samples from patients in the three groups were collected. The serum samples were stored at −80°C. The detection principle of the double‐antibody sandwich method was used with the electrochemical luminescence instrument (Roche Diagnostics GmbH, Germany).

Specific procedure: Samples of coagulant peripheral blood (8 mL) from the patients of the three groups were collected and the serum was separated by centrifugation for detection. After preheating the instrument, the “Calibration” option in the software interface (e.g., IL‐6) was selected. The instrument automatically aspirated the matching calibration samples (typically two liquids of different concentrations: Cal 1 and Cal 2). Based on the known concentrations of the calibration samples and the measured optical signals, the instrument generated a standard curve. Automated detection procedure: Sample addition and incubation (first step): The instrument probe aspirates the sample (approximately 2–5 μL) and mixes it with reagent R1 (buffer solution), then incubates the mixture at 37°C for 5 minutes to eliminate sample background interference. Incubation was performed at 37°C for approximately 9–15 min to form a “magnetic bead–antibody–antigen–antibody–ruthenium” complex. Magnetic bead incubation (second step): magnetic particles coated with streptavidin affinity reagent were added. Incubation was performed at 37°C for approximately 9 min. Magnetic separation and washing: The magnets at the chamber bottom capture the magnetic particles (complexes), whereas unbound free substances (excess antibodies and sample impurities) are aspirated and discharged by the peristaltic pump. The instrument aspirated the cleaning solution to rinse the magnetic beads and removed non‐specific adsorption. Electrochemiluminescence reaction: Injected ProCell solution (containing tripropylamine TPA). Voltage application: a specific voltage was applied to the working electrode (anode). Luminescence: Oxidoreduction reactions occurred between ruthenium complexes on the electrode surface and TPA, releasing photons (wavelength 620 nm). Signal detection: The photomultiplier tube (PMT) collected the optical signals. The light intensity is proportional to the concentrations of IL‐6/IL‐8/TNF‐α in the sample. The instrument software automatically calculates concentrations based on the standard curve.

The intra‐ and inter‐assay coefficients of variation (CV) for all these kits were < 10% and < 15%, respectively, according to the manufacturer's protocol.

### 2.6 Statistical Analysis

Statistical analyses were performed using SPSS software (version 24.0; SPSS, Chicago, IL). Non‐normally distributed continuous data are presented as median and interquartile range [M (Q)]. Comparisons of peripheral blood lymphocyte subsets, serum function markers, and cytokines among the RAU, Others, and HC groups were conducted using the Mann–Whitney U test. A *p* value of < 0.05 was considered statistically significant.

## 3 Results

### 3.1 Results of Cellular Immune Function and Comparison of Peripheral Blood Lymphocyte Subsets Among the RAU, Others, and HC Groups [M(Q)%]

**Figure 1:**
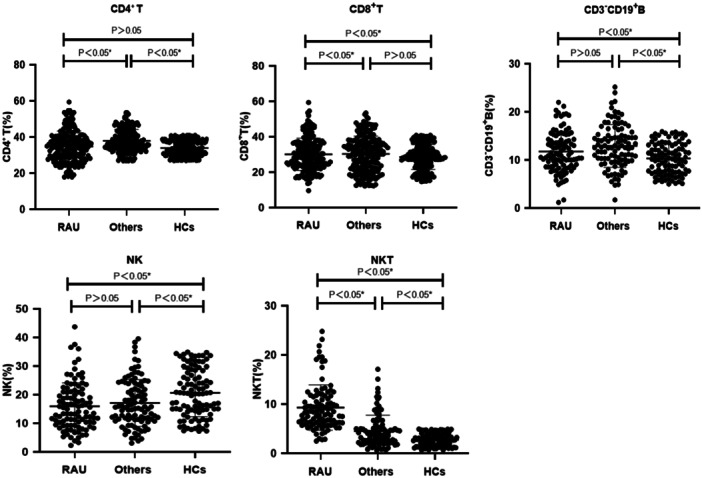
Comparison of lymphocyte subsets in RAU, Others, and HC groups.

Nonparametric tests were performed for analysis. The percentage of NKT cells in the RAU group [8.4% (5.4%)] was significantly higher than that in the Others group [3.8% (3.04%)] and the HC group [2.84% (2.32%)]; also, the percentage of NKT cells in the Others group was also significantly higher than that in the HC group. The percentages of NK cells in the RAU group [14.02% (11.05%)] and the Others group [15.7% (12.1%)] were significantly lower than that in the HC group [20.5% (13.74%)], with no statistical difference between the RAU and Others groups. The percentages of CD8 + T cells in the RAU group [24.6% (10.24%)] were significantly higher than those in the Others group [22.0% (8.9%)] and the HC group [23.81% (8.62%)], with no statistical difference between the Others and HC groups. The percentages of CD3^−^CD19 + B cells in the RAU group [11% (5.6%)] and the Others group [12.6% (5.3%)] were significantly higher than that in the HC group [10.25% (5.57%)], with no statistical difference between the RAU and Others groups (Tables 2 and 3, Figure 1).

**Table 2:** Result of immune function markers in RAU, Others, and HC groups [M(Q)].

Immune function markers		RAU group (*n* = 101)	Others group (*n* = 101)	HC group (*n* = 101)
CD4^+^T	%	34.6 (10.8)	36.7 (8.8)	33.6 (7.2)
CD8^+^T	%	24.6 (10.24)	22 (8.9)	23.81 (8.62)
CD3^‐^CD19^+^B	%	11 (5.6)	12.6 (5.3)	10.25 (5.57)
NK (CD3^‐^CD16^+^CD56^+^)	%	14.02 (11.05)	15.7 (12.1)	20.5 (13.74)
NKT (CD3^+^CD16^+^CD56^+^)	%	8.4 (5.4)	3.8 (3.04)	2.84 (2.32)
IgG	g/L	12.3 (3.75)	12.2 (2.5)	13.26 (4.07)
IgA	g/L	2.39 (1.28)	2.3 (1.27)	2.66 (1.56)
IgM	g/L	1.07 (0.61)	1.03 (0.68)	1.72 (0.99)
IgE	g/L	21.36 (4.28)	7.89 (4.54)	8.47 (11.10)
C3	g/L	0.91 (0.27)	0.87 (0.21)	1.01 (0.36)
C4	g/L	0.22 (0.11)	0.22 (0.08)	0.26 (0.17)

**Table 3:** Comparison of immune function markers in RAU, Others, and HC groups.

Immune function markers		RAU vs others		RAU vs HCs		Others vs HCs	
		Z	*p*	Z	*p*	Z	*p*
CD4^+^T	%	−2.948	0.003*	−0.360	0.719	−4.419	0.000*
CD8^+^T	%	−2.952	0.003*	−2.101	0.036*	−1.165	0.244
CD3‐CD19^+^ B	%	−1.933	0.053	−2.402	0.016*	−4.239	0.000*
NK(CD3‐CD16 + CD56 + )	%	−1.165	0.244	−3.967	0.000*	−2.963	0.003*
NKT(CD3 + CD16 + CD56 + )	%	−8.475	0.000*	−11.583	0.000*	−3.925	0.001*
IgG	g/L	−0.34	0.734	−1.148	0.251	−1.722	0.085
IgA	g/L	−0.648	0.517	−1.443	0.149	−1.957	0.050
IgM	g/L	−0.199	0.842	−5.321	0.000*	−4.669	0.000*
IgE	g/L	−6.602	0.000*	−7.422	0.000*	−0.181	0.857
C3	g/L	−1.072	0.284	−3.431	0.001*	−4.390	0.000*
C4	g/L	−0.0883	0.377	−2.072	0.038*	−2.498	0.012*

### 3.2 Results of Humoral Immune Function and Comparison of Peripheral Serum Immunoglobulins and Complement Components Among the RAU, Others, and HC Groups [M(Q) g/L]

**Figure 2:**
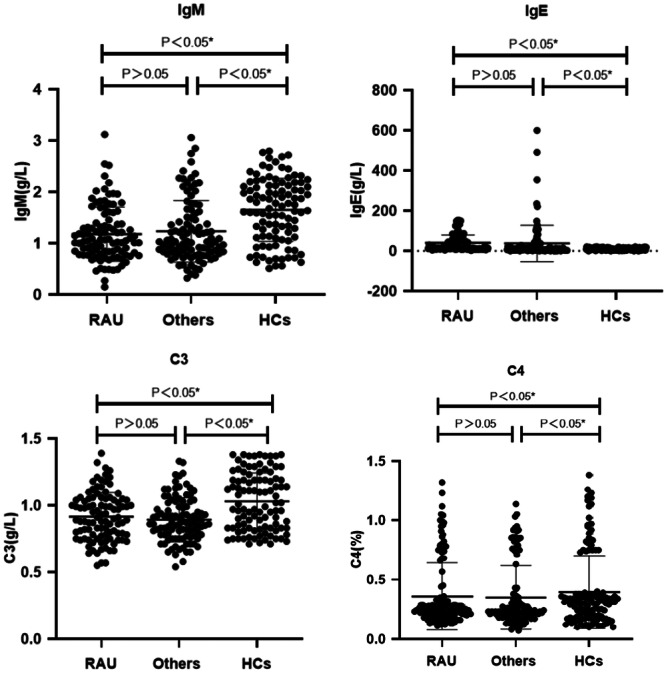
Comparison of immunoglobulin and complement in RAU, Others, and HC groups.

The concentrations of IgM in the RAU group [1.07 (0.61) g/L] and the Others group [1.03 (0.68) g/L] were significantly lower than that in the HC group [1.72 (0.99) g/L], with no statistically significant difference observed between the RAU and Others groups. The concentration of IgE in the RAU group [21.36 (4.28) g/L] was significantly higher than those in the Others group [7.89 (4.54) g/L] and the HC group [8.47 (11.10) g/L], with no statistically significant difference observed between the Others and HC groups. The concentrations of C3 and C4 in the RAU group [0.91 (0.27) g/L, 0.22 (0.11) g/L] and the Others group [0.87 (0.21) g/L, 0.22 (0.08) g/L] were significantly lower than that in the HC group [1.01 (0.36) g/L, 0.26 (0.17) g/L], respectively, with no statistically significant difference observed between the RAU and Others groups. No statistically significant differences were observed in the concentrations of IgA and IgG among the three groups (Tables 2 and 3, Figure 2).

### 3.3 Comparison of Serum Cytokines (IL‐6, IL‐8, and TNF‐α) Between RAU and HC Groups [M(Q) pg/ml]

**Figure 3:**
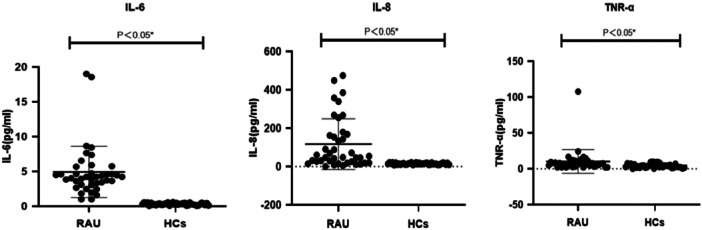
Comparison of cytokines in the RAU and HCs groups.

The concentrations of IL‐6 [4.24 (2.00) pg/mL], IL‐8 [51.50 (142.51) pg/mL], and TNF‐α [7.45 (6.35) pg/mL] in the RAU group were significantly higher than those in the HC group [0.33 (0.29), 14.72 (7.28) and 4.45 (4.96) pg/mL], respectively (Table 4 and Figure 3).

**Table 4:** Result and comparison of cytokines in RAU, Others, and HC groups [M(Q)].

Cytokines	RAU group (*n* = 40)	HCs group (*n* = 40)	RAU vs HCs	
			Z	*p*
IL‐6 (pg/mL)	4.24 (2.00)	0.33 (0.29)	−7.699	0.000*
IL‐8 (pg/mL)	51.50 (142.51)	14.72 (7.28)	−5.331	0.000*
TNF‐α (pg/mL)	7.45 (6.35)	4.45 (4.96)	−3.325	0.000*

### 3.4 Diagnostic Evaluation of Immune Function Markers and Cytokines in RAU via ROC Curve Analysis

**Figure 4:**
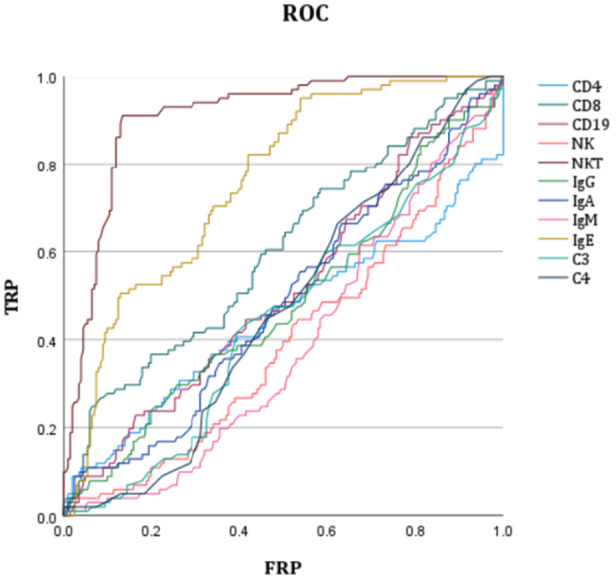
The ROC curves of NTK cell and IgE in the diagnosis of RAU were 0.908 and 0.758, respectively (*p* < 0.05).

**Figure 5:**
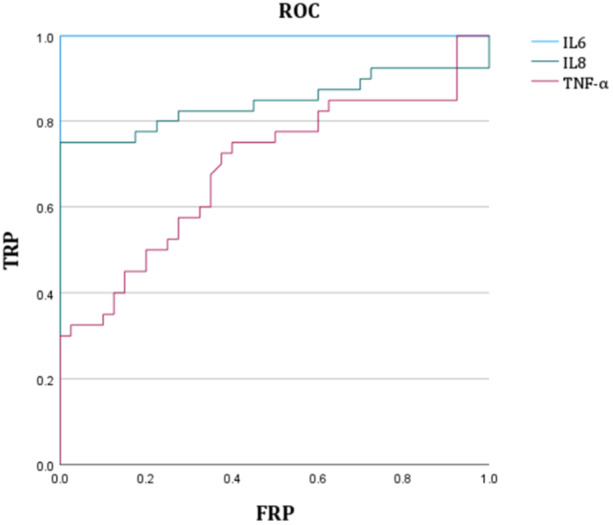
The ROC curves of IL‐6, IL‐8, and TNF‐ａ in the diagnosis of RAU were 1.0, 0.846, and 0.693, respectively (*p* < 0.05).

ROC curve analysis demonstrated that NKT cells and IgE showed AUC values of 0.908 and 0.758, respectively, in the diagnosis of RAU (both *p* < 0.05). In contrast, other lymphocyte subsets, immunoglobulins, and complement components showed AUC values below 0.7. Notably, IL‐6, IL‐8, and TNF‐α achieved AUC values of 1.0, 0.846, and 0.693, respectively, with statistical significance (all *p* < 0.05) (Table 5, Figures 4 and 5). Cytokines showed excellent diagnostic efficacy in this retrospective cohort, with IL‐6 reaching an AUC of 1.0, though this may be influenced by the limited sample size.

**Table 5:** Summary of ROC curve analysis for the investigated markers in distinguishing RAU from HCs.

Markers	AUC (95% CI)	*p*	Cut‐off values	Sensitivity (%)	Specificity (%)
NKT (%)	0.908 (0.844–0.972)	< 0.0001	> 5.75	100.0	97.5
IgE (g/L)	0.758 (0.654–0.862)	< 0.0001	> 24.91	72.0	82.5
IL‐6 (pg/mL)	1.000 (1.000–1.000)	< 0.0001	> 0.67	100.0	100.0
IL‐8 (pg/mL)	0.846 (0.782–0.910)	< 0.0001	> 24.85	92.5	97.5
TNF‐α (pg/mL)	0.693 (0.587–0.799)	< 0.0001	> 6.31	70.0	75.0

## 4 Discussion

RAU, the most prevalent oral mucosal ulcerative disease, is characterized by periodic recurrence and variable attack duration. In the absence of specific diagnostic biomarkers, diagnosis relies primarily on medical history and its distinct clinical features: yellow base, erythematous halo, central depression, and significant pain (Chinese Stomatological Association Oral Mucosal Diseases Committee 2012). Although the etiology of RAU remains elusive, immune dysregulation is widely considered a central pathogenic factor. Supporting this, studies have demonstrated an imbalance in immune cell subsets, particularly dysfunction in cellular immunity (Haibo et al. 2021; Cuiqiang et al. 2018). Consequently, evaluation of immune function through analyses of cellular lymphocyte subsets and humoral immunity can provide a valuable laboratory basis for assessing the disease course and immune status in RAU patients.

Lymphocyte subpopulations primarily consist of T lymphocytes, B lymphocytes, and natural killer (NK) cells, with NKT cells recently garnering significant attention. T lymphocytes are central to cellular immunity, characterized by the surface expression of CD3 on mature cells. These CD3^+^ T cells are further categorized into helper T cells (CD4^+^T) and cytotoxic T cells (CD8^+^T), which are pivotal in maintaining the body's cellular immune balance. Previous studies have indicated that CD3^+^, CD4^+^, and CD8^+^ T cell counts, as well as the CD4/CD8 ratio, are lower in patients with RAU and oral lichen planus compared to HCs (Hua et al. 2020; Lilin et al. 2017; Zhixiao et al. 2016; Yunyin et al. 2016). In this study, we analyzed lymphocyte subsets in both the RAU and “Other” disease groups. Our results demonstrated that the percentage of CD4^+^ T cells in the RAU group was significantly lower than that in the “Others” group; notably, the latter also showed higher levels than the HCs. Conversely, the percentages of CD8^+^ T cells in the RAU were significantly higher than those in the HC group and the “Others” group, with no statistically significant difference observed between the “Others” and HC groups. These findings suggest that helper CD4^+^ T cells are significantly suppressed in RAU patients compared to those with other oral mucosal diseases, whereas cytotoxic CD8^+^ T cells are activated in RAU patients relative to both other disease groups and HCs.

NK (CD3‐CD16 + CD56 +) cells, also known as natural killer cells, are pivotal components of innate immunity. They primarily exert cytotoxicity against target cells via perforin, NK cell–derived cytotoxic factors, and tumor necrosis factor, serving as a crucial defense line against tumors and infections (Nan et al. 2017). In this study, the percentages of NK cells in both the RAU and “Others” groups were significantly lower than those in the HC group. These findings suggest that NK cell function in RAU and other oral mucosal diseases may be compromised, thereby impairing the body's effective innate immune response.

NKT (CD3 + CD16 + CD56 + ) cells show the phenotypic and functional characteristics of both NK cells and T cells, thereby serving as a critical bridge between innate and adaptive immunity (Shimizu et al. 2020; Godfrey et al. 2018). Consequently, they have emerged as a focal point in lymphocyte subset research. Defined as innate‐like T lymphocytes with an effector/memory phenotype, these cells rapidly secrete a broad spectrum of cytokines upon activation, recruiting and activating other effector cells to participate in disease‐specific immune responses (Zhu et al. 2018). Although numerous studies have established the pivotal role of NKT cells in liver diseases (Xin et al. 2021), tuberculosis (Jie. 2021), malignancies (LI Rui 2020), and viral infections, investigations into their involvement in RAU remain scarce. In this study, the percentage of NKT cells in the RAU group was significantly higher than that in both the “Others” and HC groups; notably, the proportion in the “Others” group was also significantly elevated compared to that in the HC group. Given that NKT cells possess dual immunomodulatory functions, capable of either enhancing or suppressing immune responses, our findings indicate that they potentially play a crucial role in RAU and other oral mucosal diseases. This regulatory mechanism may be particularly significant in RAU patients in whom innate and adaptive immunity are otherwise suppressed.

B lymphocytes (CD3‐CD19 + ) are the principal mediators of humoral immunity, with CD19 serving as a critical surface antigen. Upon antigenic stimulation, B cells differentiate into effector cells that secrete antibodies to orchestrate immune responses. The specific mechanisms underlying humoral immunity in RAU remain a subject of debate; however, the existing literature suggests that humoral function is implicated in RAU and oral lichen planus, as evidenced by elevated levels of IgM, IgG, IgA, and complement components in affected patients (Haibo et al. 2021; Yunyin et al. 2016; Nan et al. 2017). In this study, the percentages of CD3‐CD19 + B cells in both the RAU and “Others” groups were significantly higher than those in the HC group, whereas no statistically significant difference was observed between the RAU and “Others” groups. These findings suggest that humoral immune cells may play an active role in the pathogenesis of RAU and other oral mucosal diseases.

Immunoglobulin M (IgM) serves as a primary mediator of the humoral immune response, possessing antibody activity that aids the host in resisting foreign pathogens. Furthermore, IgM is capable of activating the complement system. The terminal pathway of this activation mediates cell lysis; concurrently, the release of histamine and other bioactive substances during this process can trigger local inflammatory responses, potentially leading to oral mucosal ulceration (Haibo et al. 2021). In this study, the levels of IgM (along with C3 and C4) in both the RAU and “Others” groups were significantly lower than those in the HC group. These findings suggest that IgM synthesis is reduced in RAU and other oral mucosal diseases, which may lead to the suppression of complement activation and result in aberrant humoral immune function.

Immunoglobulin E (IgE) serves as the primary antibody mediating type I hypersensitivity. It binds to high‐affinity FcεRI receptors on mast cells and basophils, triggering the degranulation of these cells and the release of inflammatory mediators, such as histamine and leukotrienes, which drive allergic reactions. Previous studies have indicated that salivary IgE levels can serve as a preliminary assessment marker for patients with aphthous stomatitis (Haibo et al. 2021; Farhad‐Mollashahi et al. 2020; Assiri et al. 2023). In this study, IgE levels in the RAU group were significantly higher than those in both the “Others” and HC groups. These findings suggest that IgE potentially plays a critical role in the humoral immune dysregulation associated with RAU (Haibo et al. 2021).

Cytokines play a pivotal regulatory role in immune responses and various physiological processes. Based on their structure and function, they are categorized into interleukins (ILs), interferons (IFNs), tumor necrosis factors (TNFs), colony‐stimulating factors (CSFs), chemokines, and growth factors. Among these, interleukins and TNFs are of particular significance in the study of oral diseases (Teng and Jin 2024; Xiao et al. 2023; GAO Xiaolan 2021). In this study, we measured and compared the serum levels of IL‐6, IL‐8, and TNF‐α in patients with RAU and HCs. IL‐6 and IL‐8, members of the interleukin family, are primarily involved in immune responses and inflammatory regulation. Previous studies have indicated that elevated IL‐6 levels in RAU patients can exacerbate inflammation, thereby promoting ulcer progression (Najafi et al. 2016; Shabana et al. 2021; Liru et al. 2024). TNF‐α, mainly produced by macrophages and multinucleated giant cells, plays a critical role in regulating immune function and mediating inflammation, tissue injury, and other pathophysiological reactions such as shock. Extensive research has demonstrated that serum TNF‐α levels are significantly higher in RAU patients than in healthy individuals (Liru et al. 2024; Edmans et al. 2022; Surboyo et al. 2022). Consistently, our results showed that the serum concentrations of IL‐6, IL‐8, and TNF‐α in the RAU group were significantly higher than those in the HC group. Notably, IL‐6 achieved AUC value of 1.0; this perfect discrimination is likely attributable to the relatively small sample size of the included data. Even so, these findings still suggest that the cytokines IL‐6, IL‐8, and TNF‐α potentially play a crucial role in the pathogenesis of RAU. Due to limited sample availability, the cytokine analysis was performed on a subset of 40 paired samples (40 patients with RAU and 40 HCs). These participants were selected from the larger cohort based on strict inclusion criteria and sample quality. Patients in the “Others” group were excluded from this specific assay to focus specifically on the differential profile between active RAU and healthy mucosa.

However, this study is subject to several limitations. First, the single‐center design and the relatively small sample size may limit the generalizability of our findings; our results may not be fully representative of the broader population. Second, the retrospective nature of the study introduces the potential for selection bias. Third, this study did not assess the correlations between the investigated markers and RAU severity or recurrence frequency. Future studies are warranted to evaluate whether these biomarkers can serve as indicators for disease progression or prognosis.

## 5 Conclusion

In summary, the pathogenesis of RAU and other oral mucosal diseases is intrinsically linked to immune dysfunction. The comprehensive assessment of immune function—encompassing cellular immunity (lymphocyte subset analysis), humoral immunity (immunoglobulin and complement detection), and cytokine profiling—holds significant clinical value for diagnosing RAU and evaluating patient immune status. Furthermore, alterations in NKT cells and lymphocyte subsets, alongside humoral markers such as IgE and pro‐inflammatory cytokines (IL‐6, IL‐8, and TNF‐α), potentially offer a robust laboratory basis for monitoring the onset and progression of RAU and show promise as potential diagnostic biomarkers for RAU, although larger‐scale validation is necessary.

## Author Contributions

Y.C. and T.M. conducted the experiments. Y.C., L.Z., and Q.K. designed the study. Y.C. and T.M. wrote the manuscript. All the authors have read and approved the final manuscript.

## Ethics Statement

The Ethics Committee of Hangzhou Normal University Affiliated Hospital, China. 2019 (E02)‐HS‐04, 2020 (E2)‐HS‐06, 2023(E2)‐HS113.

## Consent

The authors have nothing to report.

## Conflicts of Interest

The authors declare no conflicts of interest.

## Publish Statement

A preprint has previously been published (Chen et al. 2025).

## Data Availability

The data that support the findings of this study are available from the corresponding author upon reasonable request.
